# Representation of Native Hawaiian and Pacific Islander Individuals in Clinical Trials

**DOI:** 10.1001/jamanetworkopen.2024.42204

**Published:** 2024-10-29

**Authors:** Deborah A. Taira, Mona Shing Ranken, Brendan K. Seto, James Davis, Andrea H. Hermosura, Cody Porter, Tetine L. Sentell, Munirih Taafaki, Julia Takata, Kauilaonalani Tengan, Connie M. Trinacty, Todd B. Seto

**Affiliations:** 1 Daniel K. Inouye College of Pharmacy, University of Hawai‘i at Hilo; 2 The Queen’s Medical Center, Honolulu, Hawai‘i; 3 Kaiser Permanente, Oakland, California; 4 John A. Burns School of Medicine, University of Hawai‘i at Mānoa, Honolulu; 5 Office of Public Health Studies, University of Hawai‘i at Mānoa, Honolulu

## Abstract

**Importance:**

Having diverse participants in clinical trials ensures new drug products work well across different demographic groups, making health care safer and more effective for everyone. Information on the extent of Native Hawaiian and Pacific Islander participation in clinical trials is limited.

**Objective:**

To examine representation of Native Hawaiian and Pacific Islanders in clinical trials leading to the first US Food and Drug Administration (FDA) approvals for the 10 drug products with the top worldwide sales forecasts in 2024.

**Design, Setting, and Participants:**

Cross-sectional secondary analysis of existing data from clinical trials that took place from 2006 to 2021 in the US. All clinical trials that were included in the FDA first approval application for the 10 drug products were evaluated in this study. Data were analyzed from February to August 2024.

**Exposure:**

Participation in a clinical drug trial.

**Main Outcomes and Measures:**

Comparison of the proportion of Native Hawaiian and Pacific Islander participation in clinical trials for the 10 drug products with top sales forecasts in 2024 to the Native Hawaiian and Pacific Islander population proportion.

**Results:**

In this cross-sectional study of 139 062 individuals, Native Hawaiian and Pacific Islander participation in clinical trials for the 10 drug products with top sales forecasts was either unknown or low. For 6 of the 10 drug products (60%), the number of Native Hawaiian and Pacific Islander participants was not documented. All trials that reported Native Hawaiian and Pacific Islander participation had fewer Native Hawaiian and Pacific Islander participants than would be expected based on their US population proportion, with 2 of the differences being statistically significant. Of the trials that disaggregated Native Hawaiian and Pacific Islander participants from other racial groups, the number of Native Hawaiian and Pacific Islander participants was 8 for risankizumab-rzaa (0.38% of participants vs 0.49% of the population; percentage point difference, −0.11%; 95% CI, −0.37% to −0.15%), 7 for bictegravir/emtricitabine/tenofovir alafenamide (0.38% of participants vs 0.49% of the population; percentage point difference, −0.10%; 95% CI, −0.39% to 0.18%), 27 for 4vHPV/9vHPV (0.15% of participants vs 0.46% of the population; percentage point difference, −0.31%; 95% CI, −0.37% to −0.26%), and 90 for BNT162B2 COVID-19 vaccine (0.20% of participants vs 0.52% of the population; percentage point difference, −0.32; 95% CI, −0.36% to −0.27%).

**Conclusions and Relevance:**

In this cross-sectional study, limited documentation and participation of Native Hawaiian and Pacific Islander individuals in clinical trials for drug products with top sales forecasts was found. This is especially concerning because Native Hawaiian and Pacific Islander individuals have a higher risk than other racial groups for type 2 diabetes, cancer, and several other conditions the products examined in this study treat. Given the importance of enrolling Native Hawaiian and Pacific Islander participants in clinical trials, sites should be established in key geographic regions, such as Hawai‘i, and postmarket studies should be conducted within Native Hawaiian and Pacific Islander populations.

## Key Points

**Question:**

Is the proportion of Native Hawaiian and Pacific Islander participants in clinical trials for the 10 drug products with top worldwide sales forecasts in 2024 different from the proportion of Native Hawaiian and Pacific Islander individuals in the US population?

**Findings:**

Iin this cross-sectional study of 139 062 individuals, clinical trials submitted for the first FDA approvals of 60% of drug products did not report the number of Native Hawaiian and Pacific Islander participants. All trials that reported Native Hawaiian and Pacific Islander participation had fewer than would be expected based on their population proportion in the US, with differences for 2 of the drug products being statistically significant.

**Meaning:**

Native Hawaiian and Pacific Islander participation in the clinical trials analyzed in this study was either unknown or minimal, suggesting that purposeful strategies for Native Hawaiian and Pacific Islander inclusion are needed, including establishing recruitment sites in key areas such as Hawai‘i with a large Native Hawaiian and Pacific Islander population.

## Teaser

This cross-sectional study evaluates representation of Native Hawaiian and Pacific Islander Individuals in clinical trials that led to the first US Food and Drug Administration approval for 10 widely used drugs.

## Introduction

Clinical trials are important to determine if a new intervention is safe, works better, and/or has fewer adverse effects than an existing treatment or intervention.^1^ To develop drug products that are safe and effective in all populations, clinical trials need to enroll a wide range of participants to ensure that vaccines and drugs work across races and ethnicities. Without equitable representation, gaps in knowledge can arise that reduce the quality of health care decision-making and the development of more effective drug products or interventions for all populations.^2^

The Native Hawaiian and Pacific Islander population in the US includes those with origins in Hawai‘i, Guam, American Samoa, the Commonwealth of the Northern Mariana Islands, the Republic of the Marshall Islands, the Republic of Palau, and the Federated States of Micronesia, as well as many others. The US Native Hawaiian and Pacific Islander population includes more than 25 groups that vary in languages, immigration and colonization experiences, and cultural traditions. According to 2021 US Census data, there were 1.7 million Native Hawaiian and Pacific Islander individuals, alone or in combination with other races, living in the US, representing about 0.5% of the US population.^3^ From 2020 to 2021, the Native Hawaiian and Pacific Islander population grew by 1.5%, which was the largest percentage increase of any racial group. In 2021, the Native Hawaiian population (683 353 individuals) was the largest detailed Native Hawaiian and Pacific Islander group in the US Census, followed by Samoan (243 682 individuals) and Chamorro (142 516 individuals) populations. These numbers are believed to undercount the actual population of these communities.^4^

In 1997, the Office of Management and Budget (OMB) revised the race standards for federal statistics and administrative reporting by separating the “Asian or Pacific Islander” category into “Asian” and “Native Hawaiian or Pacific Islander.”^5^ This mandate stated that the Native Hawaiian and Pacific Islander category be included as 1 of the 5 main racial categories as soon as possible but no later than January of 2003. While the OMB released new standards in March of 2024, the recommendation to have Native Hawaiian or Pacific Islander as a distinct category remains the same.

Numerous reviews have documented the demographic characteristics, including race and ethnicity, of clinical trial participants in general and for treatment of acute and chronic health conditions, including obesity, cancer, cardiovascular disease, glaucoma, multiple sclerosis, and infectious diseases.^6,7,8,9,10,11,12^ Recently, the US Food and Drug Administration (FDA) Center for Drug Evaluation and Research (CDER) issued a Drug Trial Snapshot Annual Report summarizing demographic information on 2023 clinical trial participants.^13^ Like most of these scoping reviews of clinical trial diversity, this 2023 FDA CDER report excludes data and discussion of clinical trial participation by Native Hawaiian and Pacific Islander individuals. An analysis of pediatric trials found Native Hawaiian and Pacific Islander individuals were significantly underrepresented (odds ratio, 0.66; 95% CI, 0.61-0.72), relative to their respective US population.^8^ In the few reviews that document Native Hawaiian and Pacific Islander participation, a striking percentage of trials had 0 Native Hawaiian and Pacific Islander participants. For instance, a review of the vaccine trials reporting race found that 60% had 0 Native Hawaiian and Pacific Islander participants.^10^ Similarly, in a review of cardiovascular device studies, while 59% detailed Native Hawaiian and Pacific Islander participation, 46% of these had 0 Native Hawaiian and Pacific Islander clinical trial enrollees.^12^

Inclusion of Native Hawaiian and Pacific Islander participants in clinical trials is particularly important because Native Hawaiian and Pacific Islander individuals are disproportionately affected by many acute and chronic conditions.^14^ Compared with non-Hispanic White populations, Native Hawaiian and Pacific Islander populations had higher rates of asthmatic episodes, cancer deaths, COVID-19 morbidity and mortality, and type 2 diabetes.^15,16,17^ The objective of this descriptive study is to examine representation of Native Hawaiian and Pacific Islander individuals in clinical trials for the first FDA approvals of drug products with the top sales forecasts in 2024.

## Methods

Since this study used deidentified, publicly available aggregate data, institutional review board approval and informed consent were not required in accordance with 45 CFR §46. This manuscript follows Strengthening the Reporting of Observational Studies in Epidemiology (STROBE) reporting guidelines.^18^

### Study Design, Setting, and Participants

This retrospective observational study used publicly available data from the FDA and the US Census Bureau. The 10 drug products (pembrolizumab,^19^ semaglutide,^20^ dupilimab,^21^ apixaban,^22^ bictegravir/emtricitabine/tenofovir alafenamide,^23^ daratumumab,^24^ nivolumab,^25^ BNT162B2 COVID-19 vaccine,^26^ 4vHPV/9vHPV,^27,28^ and risankizumab-rzaa^29^) included in this study had the top sales forecasts in 2024.^30^ Participation in clinical trials for 4vHPV and 9vHPV were combined because the study identifying the 10 drug products with the top sales forecasts combined these 2 drug products.^30^

To have a high sales forecast, a drug product would need to have high utilization, a high price, or a combination of both. In any case, these drug products with high sales forecasts represent a cost burden to society and may significantly impact health care budgets, resource allocation, and policymaking decisions. Higher-cost drug products often represent breakthrough therapies that come with significant health benefits so examining equitable representation in these trials is important.

### Variables

Table 1 lists the generic drug name, therapeutic class, indication, and sales forecast for each drug product. For this secondary analysis of existing data, race was classified in the publicly available FDA clinical or medical reviews based on data received from the sponsors of the clinical trials. As stated by the OMB, race is a sociocultural construct.^5,31,32^ Native Hawaiian and Pacific Islander populations are indigenous to island nations and territories with diverse language, culture, history, and traditions; however, these communities have shared experiences that are important to health outcomes, including significant historical trauma, colonization, systematic racism, and limited socioeconomic opportunities.^33,34,35^ Recognizing Native Hawaiian and Pacific Islander individuals as a distinct racial category acknowledges these shared experiences and helps address the unique health challenges they face, which are often shaped by their collective history and social and political factors. The FDA recommends that clinical trials use OMB guidance on reporting race and ethnicity.^36^ While this can minimize important heterogeneity within racial categories, it does provide a standardized classification to compare health-related outcomes and to track progress toward achieving health equity.

**Table 1.** Description of the 10 Drug Products With the Top Worldwide Sales Forecasts in 2024

Generic drug name	Pharmacological class	Year of first FDA approval	2024 Worldwide sales forecasts (billions, US $)^30^	First FDA approval indication
Pembrolizumab	Anti-PD-1 mAb	2014^a^	27	Cancer (melanoma)
Semaglutide	GLP-1 receptor agonist	2017	16	Type 2 diabetes
Dupilimab	Anti-IL-4/IL-13 mAb	2017	13	Eczema (atopic dermatitis)
Apixaban	Factor Xa inhibitor	2012	13	Atrial fibrillation
Bictegravir/emtricitabine/tenofovir alafenamide	HIV INSTI/NRTI/NtRTI	2018	13	HIV
Daratumumab	Anti-CD38 mAb	2015	12	Cancer (multiple myeloma)
Nivolumab	Anti-PD-1 mAb	2014^a^	11	Cancer (melanoma)
BNT162b2 COVID-19 vaccine	SARS-CoV-2 vaccine	2021	11	SARS-CoV-2 vaccine
4vHPV/9vHPV	HPV vaccine	2006 /2014	10	HPV vaccine
Risankizumab-rzaa	Anti-IL-23 mAb	2019	10	Plaque psoriasis

Abbreviations: FDA, US Food and Drug Administration; GLP-1, glucagon-like peptide-1; HPV, human papillomavirus; IL, interleukin-x; INSTI/NRTI/NtRTI, integrase strand transfer inhibitor/nucleoside reverse transcriptase inhibitor/nucleotide reverse transcriptase inhibitor; mAb, monoclonal antibody; PD-1, programmed cell death protein 1.

^a^ First FDA accelerated approval.

### Data Sources and Bias

Information on the number of Native Hawaiian and Pacific Islander participants in the clinical trial(s) that led to the first approval by the FDA for each drug product was extracted from FDA review documents publicly available on FDA websites. Bias was reduced by focusing on the 10 drug products with the top worldwide sales forecasts from a published study.^30^ The research team also used a standardized approach to obtain information for each drug product that involved using key terms to identify publicly available review documents on the FDA website.

### Outcomes and Measures

According to US Census data, Native Hawaiian and Pacific Islander individuals, alone or in combination with other races, were approximately 0.2% of the US population in 2006, 0.4% in 2012, and 0.5% in 2013 and in subsequent years.^3^ For each drug, the number of Native Hawaiian and Pacific Islander participants needed for the sample to be reflective of the population was calculated by multiplying the number of participants by the percentage of Native Hawaiian and Pacific Islander individuals in the US population for the year of initial FDA approval (between 0.2% and 0.5%). For instance, if there were 1000 clinical trial participants in 2015, 5 should be Native Hawaiian and Pacific Islander individuals if the participant sample was reflective of the population. If a drug product had more than 1 clinical trial included in the FDA clinical or medical reviews for the first FDA approvals, the number of participants enrolled in each clinical trial was summed. If some clinical trials reported the number of Native Hawaiian and Pacific Islander participants and others did not, existing data were analyzed and a not available designation was given to trials without information on Native Hawaiian and Pacific Islander participation.

### Statistical Analysis

To examine whether the proportion of Native Hawaiian and Pacific Islander individuals in the clinical trial for each drug differed at the .05 level of significance from the proportion of Native Hawaiian and Pacific Islander individuals in the US population, we used a test of proportions. Two-sided, 2-proportion *z* tests were used to compare the proportion of Native Hawaiian Pacific Islander participants in clinical trials to their proportion in the general population. Assuming a population proportion of 0.005, the minimal sample size required to detect a difference in proportions of 0.001 with 80% power and a significance level of .05 is approximately 37 883 participants. A proportional difference of 0.003 would require at least 4209 participants. All analyses were performed using Stata MP version 17 (StataCorp) from February to August 2024.

## Results

Table 2 displays the number of Native Hawaiian and Pacific Islander participants included in each clinical trial and the number that would be expected if the proportion reflected the US population. For 6 of the 10 drug products (60%), the number of Native Hawaiian and Pacific Islander participants was not documented at all. For the remaining 4 drug products (40%), the proportion of Native Hawaiian and Pacific Islander participants was less than the proportion of Native Hawaiian and Pacific Islander individuals in the US population. This difference was statistically significant for 2 of the 4 products for which the number of Native Hawaiian and Pacific Islander participants was documented. Of the trials that disaggregated Native Hawaiian and Pacific Islander individuals from other racial categories, the number of Native Hawaiian and Pacific Islander participants was 8 for risankizumab-rzaa (0.38% of participants vs 0.49% of the population; percentage point difference, −0.11%; 95% CI, −0.37% to −0.15%), 7 for bictegravir/emtricitabine/tenofovir alafenamide (0.38% of participants vs 0.49% of the population; percentage point difference, −0.10%; 95% CI, −0.39% to 0.18%), 27 for 4vHPV/9vHPV (0.15% of participants vs 0.46% of the population; percentage point difference, −0.31%; 95% CI, −0.37% to −0.26%), and 90 for BNT162B2 COVID-19 vaccine (0.20% of participants vs 0.52% of the population; percentage point difference, −0.32; 95% CI, −0.36% to −0.27%).

**Table 2.** Comparison of Proportion of Native Hawaiian and Pacific Islander Individuals Among Clinical Trial Participants for the First FDA Approval to Proportion of Native Hawaiian and Pacific Islander Individuals in the US Population for Drug Products With Top 10 Worldwide Sales Forecasts in 2024

Generic drug name	First FDA approval indication	Total participants in trials reviewed for first FDA approval	No. (%) of Native Hawaiian and Pacific Islander participants	Year of first FDA approval	No. Native Hawaiian and Pacific Islander participants needed to reflect US population at time of first FDA approval	*P* value
Pembrolizumab	Cancer (melanoma)	1577^a^	NA	2014^a^	8	NA
Semaglutide	Type 2 diabetes	8104	NA	2017	41	NA
Dupilimab	Eczema (atopic dermatitis)	2855	NA	2017	14	NA
Apixaban	Atrial fibrillation	33 150	NA	2012	133	NA
Bictegravir/emtricitabine/tenofovir alafenamide^b^	HIV	1855	7 (0.38)	2018	9	.52
		676	NA		3	NA
Daratumumab	Cancer (multiple myeloma)	331	NA	2015	2	NA
Nivolumab	Cancer (melanoma)	886^a^	NA	2014^a^	4	NA
COVID-19 vaccine, mRNA	SARS-CoV-2 vaccine	44 165	90 (0.20)	2021	221	<.001
4vHPV	HPV vaccine	27 008	NA	2006	54	NA
9vHPV^b^		18 455	27 (0.15)	2014	92	<.001
		3820	NA		19	NA
Risankizumab-rzaa^b^	Plaque psoriasis	2109	8 (0.38)	2019	11	.46
		292	NA		1	NA

Abbreviations: FDA, US Food and Drug Administration; HPV, human papillomavirus; NA, not available.

^a^ First FDA accelerated approval.

^b^ Multiple rows are included for drug products that had Native Hawaiian and Pacific Islander participation data for some but not all the trials included in the FDA reviews.

## Discussion

Ensuring Native Hawaiian and Pacific Islander representation in clinical trials for US marketing authorization approvals of drug products is essential so that Native Hawaiian and Pacific Islander individuals have access to innovative new therapies and so their unique needs are addressed in the development and evaluation of high-impact treatments. This is particularly important for Native Hawaiian and Pacific Islander populations, who are at higher risk for diseases such as type 2 diabetes and cancer, making them more likely to be prescribed treatments for these conditions.^14^ Moreover, 2 of the 10 drug products with top sales forecasts treat advanced melanoma cancer, for which Native Hawaiian and Pacific Islander patients have worse survival than non-Hispanic White patients.^37^ To better understand the representation of Native Hawaiian and Pacific Islander patients, we examined the clinical trials that led to the first FDA approval for 10 drug products with the top sales forecasts in 2024. We found that the clinical trials for 60% of the drug products examined in this study did not document Native Hawaiian and Pacific Islander participation. Among the clinical trials that did, Native Hawaiian and Pacific Islander patient enrollment was generally low and less than the Native Hawaiian and Pacific Islander proportion of the US population.

Inclusion of Native Hawaiian and Pacific Islander participants in these clinical trials allows health care clinicians to better assess the relevance of the trial data to their specific care needs, which may be affected by historical trauma, racism, colonialism, and other societal factors.^33,34,35^ Furthermore, involving Native Hawaiian and Pacific Islander participants in trials also ensures equitable access to innovative, cutting-edge therapies, which is particularly important for people with disease conditions for which effective treatments are not currently available. An inclusive approach to enrollment helps minimize data gaps and biases, promoting fairness and justice. This approach aligns with a core principle of ethical research: that its benefits should be accessible to everyone.

Despite the 1997 OMB mandate,^5^ most of the phase 3 and pivotal clinical trials for these drug products with the top sales forecasts in 2024 did not disaggregate the number of Native Hawaiian and Pacific Islander participants, often combining them with Asian participants or putting Native Hawaiian and Pacific Islander individuals into an other race category. When Native Hawaiian and Pacific Islander and Asian participants are grouped together in clinical trial reports, they are often labeled only as “Asian,” rather than “Asian and Native Hawaiian and Pacific Islander,” which both inflates the perceived percentage of Asian participants in the trial and renders Native Hawaiian and Pacific Islander participants invisible. Failure to disaggregate Native Hawaiian and Pacific Islander participant data when discussing the racial distribution of study participants not only perpetuates Native Hawaiian and Pacific Islander invisibility in clinical research, but it also implies that Native Hawaiian and Pacific Islander experiences and health outcomes are not valued.^38^ Including Native Hawaiian and Pacific Islander participants in clinical trials is essential to promoting health equity. Documenting the number, no matter how few, is important as it makes the level of inclusion transparent and enables assessment of Native Hawaiian and Pacific Islander participation relative to their population proportion.

For the clinical trials examined in this study that contained information on Native Hawaiian and Pacific Islander participation, the number of Native Hawaiian and Pacific Islander participants was lower than is reflective of the US population. For 2 of these drug products, BNT162B2 COVID-19 vaccine and 4vHPV/9vHPV, this difference reached statistical significance at the .05 level. For the other 2 products, bictegravir/emtricitabine/tenofovir alafenamide and risankizumab-rzaa, the proportion of Native Hawaiian and Pacific Islander participation were approximately 0.4%, compared with the 0.5% population proportion. Hence, these clinical trials were able to enroll Native Hawaiian and Pacific Islander participants at a rate close to the US population proportion. Further research is needed to better understand how they were able to achieve this.

In 2022, the US Congress passed the Food and Drug Omnibus Reform Act (FDORA), which included provisions designed to increase diversity in phase 3 and other pivotal clinical trials that support drug and device approvals by the FDA.^39,40^ Clinical trial sponsors are required to submit diversity action plans containing the justification for the racial distribution of participants, including characterization of the prevalence and/or incidence of the given disease in diverse racial and/or ethnic populations, and the plans for enrollment of participants from underrepresented racial and/or ethnic populations for the given disease being investigated. The goal is to have the demographic characteristics of clinical trial participants reflect the characteristics of people with the disease or condition in the US population.

Although the clinical trials examined in this study occurred before the passage of FDORA, it is now more crucial than ever for trial sponsors to separate Native Hawaiian and Pacific Islander participants from other racial groups. Additionally, they will need to have estimated prevalence and/or incidence information in diverse racial populations to fulfill the requirements of the FDORA law.

There are several reasons why Native Hawaiian and Pacific Islander individuals might not be participating in these clinical trials. One reason is lack of awareness and limited access to clinical trials. Increasing primary care physician knowledge of clinical trial opportunities and gaining their support for these trials might be one way to increase enrollment of Native Hawaiian and Pacific Islander participants.^41,42^ Another reason for lack of participation might be lack of trust in the US health care system, which could be improved by engaging Native Hawaiian and Pacific Islander community leaders as research staff, investigators, and stakeholder advisory board members.^43^ Moreover, language barriers, cultural differences, and limited health care access might limit participation.^44^

Part of the reason for lack of Native Hawaiian and Pacific Islander participation in clinical trials might also be that many Native Hawaiian and Pacific Islander individuals live in certain parts of the US, while many clinical trials enroll patients in locations outside of the US or in clinical settings in the US that do not have a large concentration of Native Hawaiian and Pacific Islander individuals. Recruiting worldwide can increase the number of patients meeting inclusion criteria to expedite recruitment; however, worldwide representation in clinical trials does not reflect characteristics of individuals in the US who might be using the drugs and devices approved by the FDA. The largest Native Hawaiian and Pacific Islander populations in the US can be found in Hawai‘i and California, but there are counties with high concentrations of Native Hawaiian and Pacific Islander individuals in Washington, Arkansas, New York, Utah, and Florida.^45^

Sponsors could establish recruitment sites in these key geographic areas, including at least 1 clinical trial recruitment site in Hawai‘i, where a significant portion of Native Hawaiian and Pacific Islander individuals reside. Establishing a sustainable site in Hawai‘i would improve accessibility and encourage local participation. A second approach would be for investigators at primary clinical trial sites to partner with investigators and/or clinical practices in diverse settings to set up a referral network to engage Native Hawaiian and Pacific Islander communities that can be found in various parts of the US to build trust and establish strong community ties. Third, adopting mobile and decentralized clinical trial models with telehealth visits, home visits, and localized health services could enhance accessibility for potential Native Hawaiian and Pacific Islander participants. Not only would this potentially increase the size of their studies, but it would also potentially diversify their population and allow access to innovative therapies to historically marginalized groups.

Even if the proportions of Native Hawaiian and Pacific Islander participants in a phase 3 or pivotal trial was reflective of the Native Hawaiian and Pacific Islander population proportion, the number of Native Hawaiian and Pacific Islander participants would still be small in many trials. Native Hawaiian and Pacific Islander individuals represent only 0.5% of the US population, and most clinical trials enroll hundreds or thousands of participants, not tens of thousands of participants.

For instance, even though the proportion of Native Hawaiian and Pacific Islander clinical trial participants for bictegravir/emtricitabine/tenofovir alafenamide and risankizumab-rzaa was approximately 0.4%, there were only 7 and 8 Native Hawaiian and Pacific Islander participants, respectively. Therefore, it is unlikely that trials will have the power to perform subgroup analyses to examine outcomes specifically for Native Hawaiian and Pacific Islander individuals. Because of this, postmarket trials focused on Native Hawaiian and Pacific Islander populations should be conducted with larger samples to ensure that new drugs and devices are safe and effective for Native Hawaiian and Pacific Islander populations.

There is no minimal trial size below which sponsors should forgo efforts to recruit Native Hawaiian and Pacific Islander participants, nor is there a number of Native Hawaiian and Pacific Islander participants so small that it should not be documented in FDA approval submission documents. Native Hawaiian and Pacific Islander participants should be separated from Asian participants so that information on both populations is not lost.

### Limitations

This study had limitations. Our findings may underestimate the number of Native Hawaiian and Pacific Islander participants that should be enrolled in a clinical trial because calculations were based on population estimates without consideration of the differences in prevalence of disease by racial categories. Because we did not consider prevalence of disease, our calculations essentially assume equal prevalence of disease across racial populations. As Native Hawaiian and Pacific Islander populations have higher prevalence of disease for many of the conditions treated by these drug products, such as type 2 diabetes and some types of cancer, our findings likely underestimate the proportion of Native Hawaiian and Pacific Islander participants who should be included in clinical trials.^14,15,16,17^ Unfortunately, we were unable to find current population-level estimates of prevalence of disease among Native Hawaiian and Pacific Islander populations for most of the conditions for which these drug products were indicated. Future research is needed to document prevalence of disease in Native Hawaiian and Pacific Islander populations to facilitate calculations of estimates of the number of Native Hawaiian and Pacific Islander participants needed in a clinical trial for the proportion of Native Hawaiian and Pacific Islander participants in a trial to reflect the US population with a specific disease or condition. Other limitations included restricting the scope to 10 drug products and the focus on FDA first approvals, as more Native Hawaiian and Pacific Islander participants might have been included in subsequent clinical trials.

## Conclusion

Although sampling a representative population does not guarantee Native Hawaiian and Pacific Islander participation in every small trial, making a deliberate effort to recruit Native Hawaiian and Pacific Islander participants ensures that a historically marginalized population is not overlooked or excluded. Equity gives meaning to clinical trial results. With equity, clinical trial participants are representative of people with the condition in the US and the results provide the average effect for people with the condition in the country. Without equity, the results are difficult to generalize beyond the study population.

## Notes

Supplement.
Data Sharing Statement
