# Supplementary material for: Representation of Native Hawaiian and Pacific Islander Individuals in Clinical Trials
Source: JAMA Netw Open. 2024 Oct 29;7(10):e2442204. doi: 10.1001/jamanetworkopen.2024.42204 (PMC11522938; doi:10.1001/jamanetworkopen.2024.42204)
Supplement: Supplement. — Data Sharing Statement [file jamanetwopen-e2442204-s001.pdf]

# Data Sharing Statement

Taira. Underrepresentation of Native Hawaiian and Pacific Islander Individuals in Clinical Trials. *JAMA Netw Open*. Published October 29, 2024.
doi:10.1001/jamanetworkopen.2024.42204

**Data**
**Data available:** Yes
**Data types:** Data (not involving human participants)
**How to access data:** The data in the study were differences in proportions. All of the data are publicly available on the FDA website and are included in tables in the manuscript.
**When available:** With publication

**Supporting Documents**
**Document types:** None

**Additional Information**
**Who can access the data:** Anyone with access to the article.
**Types of analyses:** For any purpose
**Mechanisms of data availability:** Without investigator support, because it is not needed.
